# Population Biology of *Streptococcus pneumoniae* in West Africa: Multilocus Sequence Typing of Serotypes That Exhibit Different Predisposition to Invasive Disease and Carriage

**DOI:** 10.1371/journal.pone.0053925

**Published:** 2013-01-16

**Authors:** Eric S. Donkor, Richard A. Adegbola, Brendan W. Wren, Martin Antonio

**Affiliations:** 1 Department of Pathogen Molecular Biology, London School of Hygiene and Tropical Medicine, London, United Kingdom; 2 Vaccinology Theme, Medical Research Council, Fajara, The Gambia; 3 GlaxoSmithKline Vaccines, Wavre, Belgium; 4 Department of Microbiology, University of Ghana Medical School, Accra, Ghana; University of Florida, United States of America

## Abstract

**Background:**

Little is known about the population biology of *Streptococcus pneumoniae* in developing countries, although the majority of pneumococcal infections occur in this setting. The aim of the study was to apply MLST to investigate the population biology of *S. pneumoniae* in West Africa.

**Methods:**

Seventy three invasive and carriage *S. pneumoniae* isolates from three West African countries including The Gambia, Nigeria and Ghana were investigated. The isolates covered seven serotypes (1, 3, 5, 6A, 11, 14, 23F) and were subjected to multilocus sequence typing and antibiotic susceptibility testing.

**Results:**

Overall, 50 different sequence types (STs) were identified, of which 38% (29) were novel. The most common ST was a novel clone-ST 4012 (6.5%), and some clones including STs 913, 925, 1737, 2160 and 3310 appeared to be specific to the study region. Two STs including ST 63 and ST 4012 were associated with multiple serotypes indicating a history of serotype switching. ST 63 was associated with serotypes 3 and 23F, while ST 4012 was associated with serotypes 6A and 23. eBURST analyses using the stringent 6/7 identical loci definition grouped the 50 STs into 5 clonal complexes and 65 singletons, expressing a high level of genetic diversity among the isolates. Compared to the other serotypes, serotypes 1 and 5 isolates appeared to be more clonal. Internationally recognized antibiotic resistant clones of *S. pneumoniae* were generally absent in the population investigated and the only multidrug resistant isolate identified (1/66) belong to the Pneumocococcal Epidemiology Network clone ST 63.

**Conclusions:**

The pneumococcal population in West Africa is quite divergent, and serotypes that are common in invasive disease (such as serotypes 1 and 5) are more likely to be clonal than serotypes that are common in carriage.

## Background

Bacteria exist within populations and the concept of bacterial population biology focuses on genetic differences in a bacterial population sample and the associated evolutionary mechanisms [1]. Additionally, population biology explores the relationship of invasive strains with the rest of the population, the selective forces associated with invasiveness, and the likely effects of disturbance of the populations by interventions such as antibiotics or vaccines [1]. Since its invention in 1998 [2], multilocus sequence typing (MLST) has greatly advanced our understanding about the population biology of a wide range of bacterial pathogens [2]–[5]. MLST is based on nucleotide sequence variation of house keeping genes and its principle is that for each gene, the distinct sequences present within a bacterial species are assigned as distinct alleles, which are further used to define an allelic profile or sequence type (ST) of a strain [2]–[4]. The major advantage of MLST is that sequence data is unambiguous and the STs of isolates can be compared to those in a central database via the Internet, in contrast to other typing methods which involve comparing DNA fragment sizes on gels [2], [4]. Thus the portability of MLST means it is highly useful in monitoring the population structure of a given pathogen.

*Streptococcus pneumoniae* is part of the normal bacterial flora of the upper respiratory tract, but is also one of the most virulent microbial pathogens and a major cause of several invasive and non-invasive diseases including pneumonia, meningitis, septicaemia, sinusitis and acute otitis media. There are about one million new pneumococcal infections every year, majority of which occur in the developing world where children <5 years are most affected, and the organism is responsible for 10–20% of all deaths in this age group [6]. The public health burden related to *S. pneumoniae* is heightened by the increasing resistance of the organism to essential antimicrobial drugs. Resistant strains of the organism have been reported in both developing and developed countries, and contribute to the high mortality of its diseases [1], [5]. Though pneumococcal conjugate vaccines have become available in many countries, the vaccines only cover a handful of serotypes, and it is known that the nonvaccine serotypes derive an ecological advantage from the removal of their competitors and have been increasing in carriage prevalence, disease and antibiotic resistance [1].

There are over 90 known *S. pneumoniae* serotypes, and only a few serotypes tend to be associated with invasive disease [7]. In West Africa, serotypes 1 and 5 are major causes of invasive pneumococcal disease accounting for >30% of all cases in the region [8]. Epidemiological evidence indicates that while some *S. pneumoniae* serotypes are mainly associated with invasive disease, some are associated with carriage, while some are associated with both invasive disease and carriage [8]–[13]. So far, about eight thousand *S. pneumoniae* sequence types, covering a wide range of serotypes from different geographical locations, have been described in the *S. pneumoniae* MLST database http://speumoniae.mlst.net/ [5]. However, there is little data on African isolates.

In contrast to the developed world, the population biology of *S. pneumoniae* in developing countries is poorly understood, though the majority of pneumococcal infections occur in the developing world. In Sub-Saharan Africa, some of the major studies that have investigated the pneumococcus have focussed on serotype analysis [8], [14]–[16] which is poorly discriminatory and thus provide little understanding about population biology of the organism in the region. Recently, a number of studies have applied MLST to investigate the population biology of *S. pneumoniae* in West Africa, however, most of these studies have analysed only invasive or carriage isolates covering a limited number of serotypes [17]–[21]. To obtain a better understanding of the population biology of *S. pneumoniae* in the West African region, we applied MLST to investigate a well defined collection of invasive and carriage *S. pneumoniae* isolates covering a range of serotypes that have different predisposition to invasive disease and carriage. In this study we show that that pneumococcal serotypes that are common in invasive disease such as serotypes 1 and 5 are more likely to be clonal than serotypes that are common in carriage. We also show that the pneumococcal population in West Africa is quite divergent, and internationally recognized antibiotic resistant clones of the organism are rare in The Gambia.

## Methods

### Study Isolates

The study isolates were obtained from previous pneumococcal carriage studies [9], [18] and routine hospital surveillances of invasive pneumococcal disease [8], [14] in West Africa. The isolates from these studies had been serotyped by latex agglutination [22] and stored in 15% glycerol at −80°C. Based on information from capsule serotype, isolates for the current study were selected to cover pneumococcal serotypes of varying invasive disease potential in West Africa [8], [9], [14]–[16], [18]. Seven serotypes were selected and included serotypes 1, 3, 5, 6A, 11, 14 and 23F (only isolates with of these serotypes were included in the study). In West Africa, Serotypes 1 and 5 are common in invasive disease but rarely seen in carriage; serotypes 3 and 11 are common in carriage but not in invasive disease; serotypes 6A, 14, and 23F are common in both invasive disease and carriage. The isolates were purified on 5% blood agar and confirmed to be *S. pneumoniae* by the optochin test [23]. The MRC microbiology laboratory in The Gambia, where this work was carried out, submits to the external quality assurance programme of the United Kingdom National External Quality Assessment Service [24]. The study was approved by the MRC Scientific Coordinating Committee and the Joint MRC and Gambia Government Ethics Committee. The isolates used were gathered from various laboratories and human subjects were not enrolled in the study.

### Multilocus Sequence Typing

Genomic DNA templates were prepared from pure *S. pneumoniae* cultures as described in the manufacturer's instructions (Qiagen Genomic DNA Kit, UK) and MLST was performed as previously described [3], [19]. The MLSTprocedure involved amplification of seven housekeeping genes; electrophoretic detection of the PCR products; sequencing of the PCR products; and analysis of the MLST data.

#### Polymerase Chain Reaction

Seven housekeeping genes of *S. pneumoniae* including *aroE, gdh, gki, recP, spi, xpt* and *ddl* were targeted for amplification. Each PCR reaction was carried out in a final volume of 25 µl: 2 µg DNA template, 250 µM (each) deoxynucleoside triphosphates, 2.5 mM MgCl_2_, 25 pmol of primers, and 1 U of *Taq* polymerase. PCR cycling conditions comprised 10 min hold at 94°C (denaturation); 34 cycles of 94°C for 1 min (denaturation); 55°C for 1 min (annealing); 72°C for 1 min (extension), and a final extension at 72°C for 5 min. Amplification of DNA was performed using a MJ Research PTC-225 Peltier thermal cycler. Two µl of reaction mixtures were separated by 1.0% agarose gel electrophoresis and visualized with ethidium bromide staining and UV illumination with a gel documentation system (Gel Doc 2000; Bio-Rad, UK).

#### Sequencing of PCR products

Each sequencing reaction mixture was prepared by adding 2 ul of 1–2 uM primer (forward or reverse), 2 ul of 5x BigDye buffer (blue top), 1 ul of BigDye and bringing the final volume to 10 ul with ddH_2_O. cycling conditions comprised preheating at 96°C followed by 28–30 cycles of [96°C for 20 seconds, 55–60°C (annealing temperature) for 30 seconds, 60°C for 90 seconds], and then link to a 15°C hold). The sequencing products were purified by Sephadex G-50, and about 20 ul of each sample was denatured at 95°C for 2 minutes, followed by addition of 10 ul of formamide, and then sequencing using an ABI 3730 sequencer (Applied Biosystems).

#### Sequence analysis

Sequences were edited and complementary sense and antisense fragments were aligned using the Laser Gene DNA star 7.1 software. The sequences were submitted to the MLST database website and assigned existing or novel allele type numbers and sequence type numbers defined by the database. STs were analyzed for relatedness using the eBURST v3 program (http://eburst.mlst.net).

### Antibiotic Susceptibility Testing

Antibiotic susceptibility tests were carried out on the study isolates for six antimicrobial agents including tetracycline, chloramphenicol, erythromycin, cefotaxime, penicillin and ampicillin. Susceptibility tests were done by the disc diffusion method and E-test and the results were interpreted using the breakpoint criteria of the European Committee on Antimicrobial Susceptibility Testing [25].

## Results

### Epidemiological Background of the Study Isolates

Overall, 73 isolates were obtained from three West African countries including The Gambia (68), Nigeria (3) and Ghana (2). The isolates comprised 37 invasive and 36 carriage isolates representing respectively 31% and 14% of the original isolates from which the study isolates were selected. All the carriage isolates were recovered from the nasopharynx, while invasive isolates were recovered from specimens of blood (79%), CSF (15%) lung aspirate (3%), and knee aspirate (3%). All the carriage isolates were from children, while 21/37 of the invasive isolates were from children. A proportion of 58% (42) of the study subjects were males while 42% (31) were females. Four (11%) invasive isolates comprising two each from blood and CSF were associated with case fatality; three isolates were from children while one isolate was from an adult. Epidemiological data related to the study isolates is summarized in Table 1.

**Table 1:** Provenance of *S. pneumoniae* isolates used in this study.

Country	Surveillance method	Year of study	Age range	Invasive (n)	Carriage (n)	Ref
The Gambia	Routine hospital surveillance	1996–2003	1 day-78 years	33*	–	[8]
	Carriage study	2003–2004	<24 months		35	[9]
Nigeria	Routine hospital surveillance		2–59 months	3		[14]
Ghana	Routine hospital surveillance	2006–2007	1 day-78 years	1		[18]
	Carriage study	2006–2007	<13 years		1	[18]

* four isolates were associated with case fatality.

n- number of isolates.

Ref-reference.

### *S. pneumoniae* Population Structure

**Figure 1:**
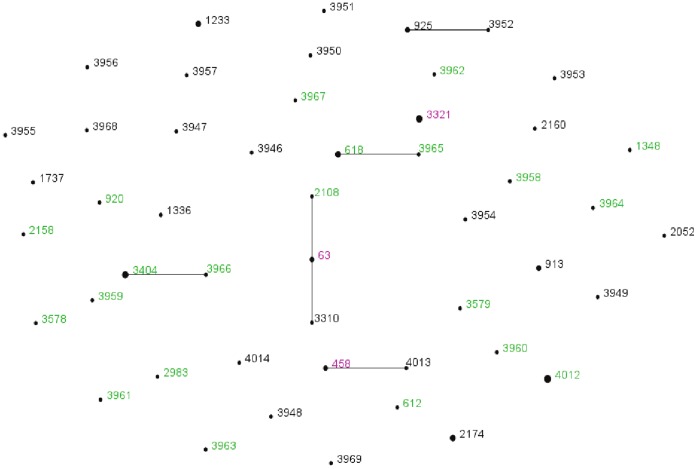
eBURST analysis of the S. pneumoniae study isolates. Each ST is represented by a point, the size of which is determined by the number of isolates with that ST in the combined data set. STs differing at a single genetic locus are linked by a straight line.

The 73 *S. pneumoniae* isolates typed by MLST yielded 51 different STs including 22 from invasive strains, 25 from carriage strains, and 3 from both types of strains. A proportion of 38% (29) of the STs were novel (i.e. they were not identified in the *S. pneumoniae* MLST database). The relatively common STs (n≥3) among invasive strains were ST4012, ST3404, ST289 and ST618, while the relatively common STs among carriage strains were ST2174 and ST1233. ST3321, ST63, and ST458 were identified among both invasive and carriage isolates. In most cases a serotype was associated with a range of STs which were usually different for invasive and carriage isolates. Two STs including ST63 and ST4012 were associated with multiple serotypes indicating a history of serotype switching. ST63 was associated with serotypes 3 and 23F, while ST4012 was associated with serotypes 6A and 23F. eBURST analyses using the stringent 6/7 identical loci definition grouped the 51 STs into 5 clonal complexes and 65 singletons, expressing a high level of genetic diversity among the strains (Figure 1). In the eBURST diagram, majority of the novel STs of the sample occurred as singletons, however six novel STs (3952, 3965, 3966, 3968, 4012, and 4013) were SLV or DLVs of previously reported STs.

### Clonal Relationship and Diversity of *S. pneumoniae* Serotypes

Serotype 1 appeared to be the most clonal serotype; 3 of the 7 serotype 1 isolates were of ST 618 while STs of the other 4 isolates (STs 612, 3579, 3960, 3965) were SLV, DLV or TLV of ST618 (Table 2). Serotype 5 also exhibited a high level of clonality, though not as serotype 1. The 15 serotype 5 isolates were of 9 STs including STs 289,1233, 3404, 3955, 3956, 3957, 3964, 3966 and 4014; the two dominant clones, ST289 and ST3404 are SLVs, while ST3966 and ST4014 are SLV and DLV of ST3404 respectively (Table 2). Serotypes 3, 6A and 14 exhibited some level of clonality but their clonal relationships were weak, and thus these serotypes especially, 3 and 6A appeared to be more heterogeneous (Table 2). Serotypes 11 and 23F did not show any clonality and were highly heterogeneous as shown by allelic profiles of their STs in Table 2.

**Table 2:** MLST of *S. pneumoniae* isolates of different serotypes.

Serotype	ST	Allelic profile											No. of isolates		
		aroE	gdh		gki		recP		spi		xpt	ddl	Invasive	Carriage	Total
Serotype 1	^+^612	10	18		4		1		7		19	31	1	0	1
	^+^618	13	8		4		1		7		19	14	3	0	3
	^+^3579	13	8		4		5		7		250	14	1	0	1
	^+^3960*	13	1		8		1		7		38	14	1	0	1
	^+^3965*	13	10		4		1		7		19	14	1	0	1
Serotype 3	63	2	5		36		12		17		21	14	0	1	1
	^×^458	2	32		9		47		6		21	17	1	2	3
	^+^925	15	16		19		15		6		20	19	0	1	1
	^+^3952*	5	16		19		15		6		20	19	0	1	1
	3953*	8	10		62		1		6		21	5	0	1	1
	3961*	1	2		83		11		13		1	7	2	0	2
	3962*	11	5		4		12		59		21	271	1	0	1
	3970*	1	1		8		18		6		3	8	1	0	1
	^×^4013*	2	32		9		47		19		21	17	0	1	1
Serotype 5	^+^289	16	12		9		1		41		33	33	3	0	3
	1233	10	11		34		16		15		1	145	0	3	3
	^+^3404	16	12		9		1		176		33	33	3	0	3
	3955*	25	31		4		1		27		28	44	0	1	1
	3956*	8	8		4		12		32		26	20	0	1	1
	3957*	11	5		1		18		6		1	5	0	1	1
	3964*	7	12		9		1		6		1	1	1	0	1
	^+^3966*	16	12		9		1		27		33	33	1	0	1
	^+^4014*	16	12		9		1		176		40	1	0	1	1
Serotype 6A	^+^913	6	57		83		28		7		19	9	0	2	2
	^+^1737	6	57		34		28		6		1	9	0	1	1
	2983	5		6		1		2		6	1	271	1	0	1
	3578	1		8		193		5		6	58	8	1	0	1
	3946*	11		8		1		5		42	1	14	0	1	1
	^+^3958*	6		57		53		28		9	19	9	1	0	1
	3959*	5		6		1		1		19	1	271	1	0	1
	4012*	7		13		53		1		72	38	31	1	0	1
Serotype 11	2052	8		5		36		3		6	1	5	0	1	1
	2158	5		5		8		1		9	1	31	1	0	1
	3947*	8		5		15		1		9	88	14	0	1	1
	3948*	118		10		4		12		17	23	6	0	1	1
	3949*	12		5		1		1		6	1	44	0	1	1
	3950*	5		5		6		1		6	1	19	0	1	1
	3951*	1		5		1		1		36	12	271	0	1	1
Serotype 14	^+^2108	11		5		36		12		17	21	14	1	0	1
	^+^3310	2		5		36		12		27	21	14	0	1	1
	^×^3321	6		60		4		5		27	20	6	2	2	4
	3963*	26		15		15		14		72	16	19	1	0	1
	^×^3967*	6		60		4		5		27	1	5	1	0	1
	3968*	11		11		4		1		6	112	14	0	1	1
**Serotype**	**ST**	**Allelic profile**											**No. of isolates**		
		**aroE**	**gdh**		**gki**		**recP**		**spi**		**xpt**	**ddl**	**Invasive**	**Carriage**	**Total**
Serotype 23F	63	2		5		36		12		17	21	14	1	0	1
	1336	7		5		4		1		6	1	14	0	1	1
	2160	6		5		5		5		27	3	5	0	3	3
	2174	7		16		8		8		6	142	14	0	4	4
	3969*	5		4		2		4		4	1	1	0	1	1
	4012*	7		13		53		1		72	38	31	4	0	4
**Total**													**37**	**36**	**73**

* indicates novel sequence type; within a serotype, STs of the same clonal complex are shown by “+” or “×”; a clonal cluster represent STs that are related as single, double or triple locus variants.

### Antibiotic Resistance

Susceptibility testing was carried out on 66 of the 73 study isolates and showed that pneumococcal resistance was highest for tetracycline (48%), followed by chloramphenicol and penicillin, both with resistance rates of 5%. No resistance was observed for erythromycin, cefotaxime and ampicillin. The prevalence of multidrug resistance (MDR) ie resistance to three or more drugs, was 2% (1/66); the only MDR isolate identified belong to the PMEN clone ST 63 and was resistant to penicillin, chloramphenicol and tetracycline. No other PMEN clones were present in the population sample, rather SLVs of two PMEN clones were identified. These included ST 612 (SLV of the PMEN clone ST 217) and ST 925 (SLV of the PMEN clone ST 236). The ST 925 strain was resistant to tetracycline but susceptible to erythromycin, cefotaxime, penicillin and ampicillin. The ST 612 strain was susceptible to all the five antibiotics tested.

## Discussion

In this study, we investigated the population biology of *S. pneumoniae* in West Africa, a region where pneumococcal outbreaks are relatively common. Though the overall sample is more representative of The Gambia (where 93% of the isolates were obtained), the serotypes selected reflect the different major pneumococcal serotypes that are common in invasive disease and/or carriage in West Africa. By comparison, some of the major clones (STs 63, 618, and 3404) reported previously in The Gambia [17], [19] were also observed among the Gambian isolates in the current study, and the relationship between these clones and serotype were consistent. The ST289 clone (serotype 5) previously reported in The Gambia [19] could be identified among the Nigerian isolates in this study rather than the Gambian isolates. Comparison of our data with that of a Ghanaian study [20] showed that two clones including ST458 (serotype 3) and ST612 (serotype 1) were identified in the Ghanaian study, as well as among our Gambian isolates. The two main clones identified in the Ghanaian study [20], namely ST217 and ST303 which were both associated with serotype 1, were not observed in the current study. However, ST618 (serotype 1) and ST612 (serotype 1) identified among our Gambian isolates are DLVs of ST217 and ST303 respectively. The Ghanaian isolates in our study sample were of two novel STs (3970 and 3969), and by comparison, neither their SLVs nor DLVs had been previously reported in Ghana. Comparison of our data with that of a recent Nigerian study [21] shows that several of the major clones in both studies are rather related as SLVs or DLVs; for example the most common clone in the current study, ST4012 is a SLV of ST802 which was the most prevalent clone in the Nigerian study.

Apart from published work, the global MLST database serves as a source of comparison for the current study. The MLST database may contain information on some STs that are absent in published papers and it also permits us to find out whether a ST or lineage is common enough to have been found. Comparison of our data with the *S. pneumoniae* MLST database [5] indicates that some clones including 913, 925, 1737, 2160 and 3310 appear to be peculiar to The Gambia, which highlights the adaptation of pneumococcal lineages to specific geographical settings. The high proportion (40%) of novel STs in this study based on comparison with the MLST database, has also been reported in other pneumococcal samples from West Africa [17]–[19]. Our West African data provide evidence of capsular switching, several of which have not been reported in the *S. pneumoniae* MLST database [5]. These observations indicate that the pneumococcal genetic background of the study region may be quite different from other geographical regions, in particular the developed world which represent majority of the MLST database.

Traditionally, serotyping had been used in typing and investigating the epidemiology of *S. pneumoniae* in West Africa. However, the association of a pneumococcal serotype with a wide range of different STs observed in this study and other studies [17]–[21], [26]–[29] demonstrates the inadequacy of serotyping in pneumococcal epidemiology. MLST is an internationally accepted method for monitoring the spread of clones through the pneumococcal population [5]. The variety of STs associated with almost every serotype in this study has been previously reported [17], [19], [20], [30], [31] and reflects the high degree of fluidity in the pneumococcal population dynamics. Our data shows that serotypes such as 1 and 5 which are common in invasive disease are more likely to be genetically homogeneous, while serotypes which are common in carriage, such as serotypes 3 and 11 tend to be genetically heterogeneous. In the case of serotype 1, this observation has been previously reported in Southern Israel [32]. Consistent with our data, serotypes 6A and 23F isolates from The Netherlands were observed to be genetically heterogeneous [33]. Recombination which is the dominant pneumococcal evolutionary mechanism is mainly associated with pneumococcal carriage [34]–[36]. Serotypes common in invasive disease are rarely carried, and are thus less exposed to recombinational events as serotypes common in carriage. Consequently, while serotypes common in invasive disease tend to be genetically homogeneous, serotypes common in carriage tend to be genetically heterogeneous. In contrast with our data, serotype 3 isolates from The Netherlands, Canada and The UK have been found to be genetically homogeneous [33], [37], [38]. This may be partly due to differences in the epidemiology of serotype 3 in West Africa and these Western Countries. While serotype 3 appears to be more common in carriage, as well as rare in invasive disease in West Africa [8], [14]–[16], it has been shown to have a high attack rate in some western countries [39], [40].

Some of the study isolates had lost viability and antibiotic susceptibility testing could be carried out on 66/73 isolates which were all from The Gambia. Tetracycline was the most resistant drug and the high prevalence of resistance observed (>50%) has been previously reported in The Gambia [19], and some other West African countries such as Ghana [18] and Nigeria [14]. Significant pneumococcal resistance to chloramphenicol was also observed though not as high as tetracycline resistance. Prevalence of chloramphenicol-resistant pneumococci in West Africa and related developing areas is reported to be 4–12% [14], [18], [19], which concurs with the 5% prevalence observed in the current study. Resistance to beta-lactam antibiotics including penicillin, ampicillin and cefotaxime was very low (<2%) which concurs with previous findings in The Gambia [19] and Mali [41]. In The Gambia as in many African countries, there is a high rate of antibiotic misuse as antibiotics are obtained over the counter without prescription [42]. It is interesting that prevalence of multidrug resistance (MDR) in a region of high antibiotic selective pressure such as The Gambia was very low (2%) which concurs with a previous study in The Gambia [19]. By comparison, MDR rates >20% have been reported in West African countries like Ghana [18] and Ivory Coast (Kacou-N'douba *et al*., 2004), while MDR rates of 5–7% have been reported in Israeli Bedouin region [43] and also Venezuelan indigenous Warao region [44]. Thus pneumococcal MDR rates in The Gambia appear to be one of the lowest in the developing world. Comparison of resistance data in this study with that of studies mentioned above, indicates that the low prevalence of pneumococcal MDR in The Gambia correlated with low resistance to beta-lactam antibiotics and erythromycin. The overall population of antibiotic resistant clones is known to be dominated by a small number of successful clones such as STs 63, 81 and 273 [45], [46]. In this study such known resistant clones were generally absent, and the single isolate which showed MDR belonged to one of such clones (ST 63). Thus it stands to reason that the very low representation of successful resistant clones in The Gambia, may be responsible for the low MDR observed. This also implies that, for the pneumococcus, selective pressure associated with widespread use of antibiotics may not be the main factor driving antibiotic resistance, but the dissemination of epidemiologically significant resistant clones.

The current study is unique in that it is based on an equal mix of carriage and invasive isolates that represent the diversity of *S. pneumoniae* in West African that has been previously reported. It may be concluded that the pneumococcal population structure investigated in this study shares some similarity with other pneumococcal population samples in West African but contrast with pneumococcal population samples from other geographical regions. The implications of these observations mean that pneumococcal response to interventions such as mass vaccination in the study region is likely to follow a different pattern from what is known in the developed world such as the United States and United Kingdom where pneumococcal vaccines have been introduced for sometime now. Pneumococcal serotypes that are common in invasive disease such as serotypes 1 and 5 are more likely to be clonal than serotypes that are common in carriage. It is also concluded that, internationally recognized antibiotic resistant clones of *S. pneumoniae* are generally absent in The Gambia and this may explain the very low occurrence of MDR strains in the country.

## References

[1] Spratt BG, Hanage WP, Brueggemann AB (2004) Evolutionary and population biology of Streptococcus pneumoniae, p.119–135 *In* Tuomanen EI, Mitchell TJ, Morrison DA, Spratt BG, editors. The pneumococcus. ASM Press, Washington, DC.

[2] Maiden MC, Bygraves JA, Feil E, Morelli G, Russell JE, et al (1988) Multilocus sequence typing: a portable approach to the identification of clones within populations of pathogenic microorganisms. Proc Natl Acad Sci USA 95 (6): 3140–3145. 10.1073/pnas.95.6.3140 PMC19708 9501229

[3] Enright MC, Spratt BG (1998) A multilocus sequence typing scheme for Streptococcus pneumoniae: identification of clones associated with serious invasive disease. Microbiology 144 (11): 3049–3060. 10.1099/00221287-144-11-3049 9846740

[4] Spratt BG (1999) Multilocus sequence typing: molecular typing of bacterial pathogens in an era of rapid DNA sequencing and the internet. Curr Opin Microbiol 2: 312–316. 10383857 10.1016/S1369-5274(99)80054-X

[5] MLST website. Available: http://spneumoniae.mlst.net/. Accessed 2012 May 26.

[6] O'Brien KL, Wolfson LJ, Watt JP, Henkle E, Deloria-Knoll M (2009) Burden of disease caused by *Streptococcus pneumoniae* in children younger than 5 years: global estimates. Lancet 374: 893–902. 19748398 10.1016/S0140-6736(09)61204-6

[7] Johnson HL, Deloria-Knoll M, Levine OS, Stoszek SK, Freimanis HL, et al. (2010) Systematic evaluation of serotypes causing invasive pneumococcal disease among children under five: the pneumococcal global serotype project. PLoS Med 7(10): pii: e1000348. 10.1371/journal.pmed.1000348 PMC2950132 20957191

[8] Adegbola RA, Hill PC, Secka O, Ikumapayi UN, Lahai G, et al (2006) Serotype and antimicrobial susceptibility patterns of isolates of Streptococcus pneumoniae causing invasive disease in The Gambia 1996–2003. Trop Med Int Health 11: 1128–1135. 16827713 10.1111/j.1365-3156.2006.01652.x

[9] Hill PC, Cheung YB, Akisanya A, Sankareh K, Lahai G, et al (2008) Nasopharyngeal carriage of Streptococcus pneumoniae in Gambian infants: a longitudinal study. Clin Infect Dis 46(6): 807–814. 18279039 10.1086/528688

[10] Obaro S (2001) Differences in invasive pneumococcal serotypes. Lancet 357: 1800–1801. 10.1016/S0140-6736(00)04915-1 11407384

[11] Brueggemann AB, Spratt BG (2003) Geographic distribution and clonal diversity of Streptococcus pneumoniae serotype 1 isolates. J Clin Microbiol 41: 4966–4970. 14605125 10.1128/JCM.41.11.4966-4970.2003 PMC262517

[12] Hausdorff WP (2007) The roles of pneumococcal serotypes 1 and 5 in paediatric invasive disease. Vaccine 25: 2406–2412. 17055620 10.1016/j.vaccine.2006.09.009

[13] Hausdorff WP, Bryant J, Kloek C, Paradiso PR, Siber GR (2000) The contribution of specific pneumococcal serogroups to different disease manifestations: implications for conjugate vaccine formulation and use, part II. Clin Infect Dis 30: 122–140. 10619741 10.1086/313609

[14] Falade AG, Lagunju IA, Bakare RA, Odekanmi AA, Adegbola RA (2009) Invasive pneumococcal disease in children aged <5 years admitted to 3 urban hospitals in Ibadan, Nigeria. Clin Infect Dis, 48 (2) 190–196. 10.1086/596500 19191615

[15] Cadoz M, Denis F, Mar ID (1981) An epidemiological study of purulent meningitis cases admitted to hospital in Dakar, 1970–1979. Bull World Health Organ 59: 575–584. 6976227 PMC2396091

[16] Holliman RE, Liddy H, Johnson JD, Ohene A (2007) Epidemiology of invasive pneumococcal disease in Kumasi, Ghana. Trans R Soc Trop Med Hyg 101: 405–413. 17126867 10.1016/j.trstmh.2006.08.014

[17] Antonio M, Hakeem I, Awine T, Secka O, Sankareh K, et al (2008) Seasonality and outbreak of a predominant Streptococcus pneumoniae serotype 1 clone from The Gambia: expansion of ST217 hypervirulent clonal complex in West Africa. BMC Microbiol 8: 198–198. 19014613 10.1186/1471-2180-8-198 PMC2587476

[18] Donkor ES, Newman MJ, Oliver-Commey J, Bannerman E, Dayie NTKD, et al (2001) Invasive disease and paediatric carriage of Streptococcus pneumoniae in Ghana. Scand J Infect Dis 42: 254–259. 10.3109/00365540903490000 20085428

[19] Antonio M, Dada-Adegbola H, Biney E, Awine T, O'callaghan J (2008) Molecular epidemiology of pneumococci obtained from Gambian children aged 2–29 months with invasive pneumococcal disease during a trial of a 9-valent pneumococcal conjugate vaccine. BMC Infect Dis, 8, 81–81. 10.1186/1471-2334-8-81 PMC2440749 18547404

[20] Leimkugel J, Adams FA, Gagneux S, Pfluger V, Flierl C, et al (2005) An outbreak of serotype 1 *Streptococcus pneumoniae* meningitis in northern Ghana with features that are characteristic of Neisseria meningitidis meningitis epidemics. J Infect Dis 192: 192–199. 15962213 10.1086/431151

[21] Adetifa IMO, Antonio M, Okoromah CAN, Ebruke C, Inem V, et al (2012) Pre-Vaccination Nasopharyngeal Pneumococcal Carriage in a Nigerian Population: Epidemiology and Population Biology. PLoS ONE 7(1): e30548 doi:10.1371/journal.pone.0030548 22291984 10.1371/journal.pone.0030548 PMC3265474

[22] Slotved HC, Kaltoft M, Skovsted IC, Kerrn MB, Spersen F (2004) Simple, rapid latex agglutination test for serotyping of pneumococci (Pneumotest-Latex). J Clin Microbiol 42: 2518–2522. 15184429 10.1128/JCM.42.6.2518-2522.2004 PMC427861

[23] Bowers EF, Jeffries IR (1955) Optochin in the identification of str. pneumoniae. J Clin Pathol 8: 58–60. 14354032 10.1136/jcp.8.1.58 PMC1023726

[24] United Kingdom National External Quality Assessment Service website. Available: http://www.ukneqas.org.uk/. Accessed 2012 May 26.

[25] The European Committee on Antimicrobial Susceptibility Testing website. Available: http://www.srga.org/eucastwt/MICTAB/index.html. Accessed 2012 May 26.

[26] Blossom DB, Cordeiro SM, Bajaksouzian S, Joloba M L, Kityo C, et al. (2007) Characterization of penicillin intermediate serotypes of Streptococcus pneumoniae carried by human immunodeficiency virus-infected adults and healthy children in Uganda. Microb Drug Resist, 13 (1), 21–28. 10.1089/mdr.2006.9993 17536930

[27] Smith-Vaughan H, Marsh R, Mackenzie G, Fisher J, Morris PS (2009) Age-specific cluster of cases of serotype 1 Streptococcus pneumoniae carriage in remote indigenous communities in Australia. Clin Vaccine Immunol 16(2): 218–221. 19091995 10.1128/CVI.00283-08 PMC2643542

[28] Marsh RL, Smith-Vaughan H, Beissbarth J, Hare K, Kennedy M, et al (2007) Molecular characterisation of pneumococcal serotype 16F: Established predominant carriage and otitis media serotype in the 7vPCV era. Vaccine 25(13): 2434–2436. 17028080 10.1016/j.vaccine.2006.09.016

[29] Mars R, Smith-Vaughan H, Hare KM, Binks M, Kong F (2010) The nonserotypeable pneumococcus: phenotypic dynamics in the era of anticapsular vaccines. J Clin Microbiol 48: 831–835. 20042626 10.1128/JCM.01701-09 PMC2832454

[30] Brueggemann AB, Griffiths DT, Meats E, Peto T, Crook DW (2003) Clonal relationships between invasive and carriage Streptococcus pneumoniae and serotype- and clone-specific differences in invasive disease potential. J Infect Dis 187: 1424–1432. 12717624 10.1086/374624

[31] Brueggemann AB, Spratt BG (2003) Geographic distribution and clonal diversity of Streptococcus pneumoniae serotype 1 isolates. J Clin Microbiol 41: 4966–4970. 14605125 10.1128/JCM.41.11.4966-4970.2003 PMC262517

[32] Porat N, Trefler R, Dagan R (2001) Persistence of two invasive Streptococcus pneumoniae clones of serotypes 1 and 5 in comparison to that of multiple clones of serotypes 6B and 23F among children in southern Israel. J Clin Microbiol 39: 1827–1832. 11325998 10.1128/JCM.39.5.1827-1832.2001 PMC88033

[33] Overweg K, Bogaert D, Sluijter M, Yother J, Dankert J, et al (2000) Genetic relatedness within serotypes of penicillin-susceptible *Streptococcus pneumoniae* isolates. J Clin Microbiol 38: 4548–53. 11101594 10.1128/jcm.38.12.4548-4553.2000 PMC87635

[34] Donkor ES, Bishop CJ, Antonio M, Wren B, Hanage WP (2011) High levels of recombination among *Streptococcus pneumoniae* isolates from the Gambia. mBio 2(3): e00040–11. 21693638 10.1128/mBio.00040-11 PMC3119534

[35] Croucher NJ, Harris SR, Fraser C, Quail MA, Burton J, et al (2011) Rapid pneumococcal evolution in response to clinical interventions. *Science* 331 (6016): 430–434. 10.1126/science.1198545 PMC3648787 21273480

[36] Feil EJ, Smith JM, Enright MC, Spratt BG (2000) Estimating recombinational parameters in Streptococcus pneumoniae from multilocus sequence typing data. Genetics 154: 1439–1450. 10747043 10.1093/genetics/154.4.1439 PMC1461021

[37] Hall LM, Whiley RA, Duke B, George RC, Efstratiou A (1996) Genetic relatedness within and between serotypes of *Streptococcus pneumoniae* from the United Kingdom: analysis of multilocus enzyme electrophoresis, pulsed-field gel electrophoresis, and antimicrobial resistance patterns. J Clin Microbiol 34: 853–859. 8815096 10.1128/jcm.34.4.853-859.1996 PMC228905

[38] Louie M, Louie L, Papia G, Talbot J, Lovgren M (1999) Molecular analysis of the genetic variation among penicillin-susceptible and penicillin-resistant *Streptococcus pneumoniae* serotypes in Canada. J Infect Dis 179: 892–900. 10068584 10.1086/314664

[39] Mufson MA, Kruss DM, Wasil RE, Metzger WI (1974) Capsular types and outcome of bacteremic pneumococcal disease in the antibiotic era. Arch Intern. Med. 134: 505–510. 4152800

[40] Martin DR, Brett MS (1996) Pneumococci causing invasive disease in New Zealand, 1987–94: serogroup and serotype coverage and antibiotic resistances. N Z Med J 109: 288–290. 8773670

[41] Yaro S, Lourd M, Traore Y, Njanpop-Lafourcade BM, Sawadogo A, et al (2006) Epidemiological and molecular characteristics of a highly lethal pneumococcal meningitis epidemic in Burkina Faso. Clin Infect Dis 43: 693–700. 16912941 10.1086/506940

[42] Vialle-Valentin CE, LeCates RF, Zhang F, Desta A, Ross-Degnan D (2012) Predictors of antibiotic use in African communities: evidence from medicines household surveys in five countries. Trop Med Int Health 17(2): 211–222. 21999394 10.1111/j.1365-3156.2011.02895.x

[43] Fraser D, Givon-Lavi N, Bilenko N, Dagan R (2001) A decade (1989–1998) of paediatric invasive pneumococcal disease in 2 populations residing in 1 geographic location: implications for vaccine choice. Clin Infect Dis 33(4): 421–427. 11462175 10.1086/321874

[44] Rivera-Olivero IA, Bogaert D, Bello T, Del Nogal B, Sluijter M, et al (2009) Pneumococcal carriage among indigenous Warao children in Venezuela: serotypes, susceptibility patterns, and molecular epidemiology. Clin Infect Dis 45 (11): 1427–1434. 10.1086/522984 17990224

[45] Sá-Leão R, Tomasz A, Sanches IS, Brito-Avô A, Vilhelmsson SE, et al (2000) Carriage of internationally spread clones of *Streptococcus pneumoniae* with unusual drug resistance patterns in children attending day care centers in Lisbon, Portugal. J. Infect. Dis 182: 1153–1160. 10.1086/315813 10979912

[46] Syrogiannopoulos GA, Grivea IN, Beratis NG, Spiliopoulou AE, Fasola EL, et al (1997) Resistance patterns of streptococcus pneumoniae from carriers attending day-care centers in southwestern Greece. Clin Infect Dis 25(2): 188–194. 9332508 10.1086/514526

